# Serum high mobility group box-1 and osteoprotegerin levels are associated with peripheral arterial disease and critical limb ischemia in type 2 diabetic subjects

**DOI:** 10.1186/s12933-017-0581-z

**Published:** 2017-08-08

**Authors:** Silvia Giovannini, Giovanni Tinelli, Federico Biscetti, Giuseppe Straface, Flavia Angelini, Dario Pitocco, Luciana Mucci, Raffaele Landolfi, Andrea Flex

**Affiliations:** 1 Department of Gerontology and Geriatrics, A. Gemelli Foundation, Catholic University of the Sacred Heart, School of Medicine, Rome, Italy; 2 Department of Vascular Surgery, A. Gemelli Foundation, Catholic University of the Sacred Heart, School of Medicine, Rome, Italy; 3 Rheumatology and Affine Sciences Institute, A. Gemelli Foundation, Catholic University of the Sacred Heart, School of Medicine, Rome, Italy; 4 Laboratory of Vascular Biology and Genetics, Catholic University School of Medicine, Rome, Italy; 5 Vascular Medicine and Atherothrombosis Laboratory, Department of Experimental Medicine, Sapienza University of Rome, Polo Pontino, Italy; 6 Department of Medicine, A. Gemelli Foundation, Catholic University School of Medicine, Rome, Italy

**Keywords:** HMGB-1, OPG, PAD, Type 2 diabetes

## Abstract

**Background:**

High mobility group box-1 (HMGB-1) is a nuclear protein also acting as inflammatory mediator, whilst osteoprotegerin (OPG), member of tumor necrosis factor receptor superfamily, is indicated as marker of vascular calcification. Peripheral artery disease (PAD) and type 2 diabetes (T2D) are clinical conditions characterized by elevated serum inflammatory markers and vascular calcification enhancement. The aim of this study was to investigate the potential role of HMGB-1, OPG and several inflammatory mediators such as C-reactive protein (HsCRP), tumor necrosis factor-alpha and interleukin-6 (IL-6) on the presence and severity of peripheral artery disease in patients with T2D.

**Methods:**

In this retrospective observational study, we have analyzed HMGB-1, OPG and inflammatory cytokines serum levels in 1393 type 2 diabetic patients with PAD and without PAD (WPAD).

**Results:**

HMGB-1 (7.89 ± 15.23 ng/mL), OPG (6.54 ± 7.76 pmol/L), HsCRP (15.6 ± 14.4 mg/L) and IL-6 (56.1 ± 28.6 pg/mL) serum levels were significantly higher in patients with PAD than in those WPAD (3.02 ± 8.12 ng/mL, *P* *˂* 0.001; 2.98 ± 2.01 pmol/L, *P* < 0.001; 7.05 ± 4.4 mg/L, P < 0.001; 37.5 ± 20.2 pg/mL, *P* < 0.001 respectively). Moreover HMGB-1 (*P* < 0.001), OPG (*P* < 0.001), HsCRP (*P* < 0.001) and IL-6 (*P* < 0.001) serum levels were positively correlated with clinical severity of PAD. HMGB-1 (adjusted OR 12.32; 95% CI 3.56–23.54, *P* = 0.023) and OPG (adjusted OR 3.53; 95% CI 1.54–6.15, *P* = 0.019) resulted independent determinants of PAD in patients with T2D after adjusting for the conventional cardiovascular risk factor and established inflammatory mediators.

**Conclusions:**

In T2D patients HMGB-1 and OPG serum levels are higher in patients affected by PAD and independently associated with its occurrence and clinical severity.

## Background

Peripheral arterial disease (PAD) is a clinical manifestation of systemic atherosclerosis affecting ten millions of people worldwide. The frequency of PAD seems to be increasing rapidly in in low-income and middle-income countries, and traditional cardiovascular risk factors of diabetes, age, smoking, dyslipidemia, and hypertension are likely to be the principal risk factors driving the epidemiological transition [1]. Atherosclerosis is now understood to be an inflammatory disease [2]. Particularly in patients with type 2 diabetes mellitus (T2D) the main mechanisms supposed involved in atherosclerotic disease progression are the decrease of nitric oxide production, the enhancement of oxidative stress, and the impairment of endothelial progenitor cell function [3]. Also in T2D inflammatory circulating molecules as C-reactive protein (HsCRP), tumor necrosis factor-alpha (TNF-alpha), and interleukin-6 (IL-6), together with abnormal endothelial expression of intercellular adhesion molecule-1 (ICAM-1) and vascular cell adhesion molecule-1 (VCAM-1) support a low-grade chronic state of inflammation and contribute to progression of disease and its complications providing prognostic information on clinical outcomes [4].

Several studies have shown that high mobility group box-1 (HMGB-1), a nuclear protein regulating gene expression, induces an inflammatory response during vascular damage [5]. HMGB-1 is a nuclear protein that has not only a role in the regulation of gene expression but also can activate pro-inflammatory responses after being passively released by damaged and necrotic cells or actively secreted by stimulated conditional innate immune cells such as endothelial cells [6, 7]. Moreover the release of HMGB-1 induced by HsCRP, one of the most sensitive biomarker of inflammation, induces, amplifies, and extends the inflammatory processes surrounding the atherosclerotic lesions [8]. Further HMGB-1 is not only released in response to pro-inflammatory stimuli, but itself causes the secretion of inflammatory molecules such as IL-6 and TNF-alpha by neutrophils and macrophages [9]. HMGB-1 has been shown be an inflammatory mediator promoting chronic inflammation and neovascularization in diabetes [10]. In addition, an associations between level of HMGB-1 and clinical complications of diabetes has been reported. Particularly a recent study showed that serum HMGB-1 is positively related to HbA1c level and is an independent predictor for coronary artery disease in patients with diabetes [11]. Moreover elevated expression of this protein has been reported in the retinas of diabetic patients and in diabetic nephropathy [12, 13]. All together those findings suggest that HGMB-1 may contribute to inflammation, endothelial dysfunction and atherosclerosis progression in diabetes mellitus [14]. Also HMGB-1 increases osteoprotegerin (OPG) expression in osteoblasts and is chemotactic to osteoclasts and osteoblasts during endochondral ossification, such as to monocytes and other immune and non-immune cells [15]. OPG is a member of the tumor necrosis factor (TNF) receptor family implied in bone turnover process, osteoporosis and premature vessel calcification [16]. Interestingly recent studies indicate that OPG is an important regulatory molecule in vascular diseases, including cerebral atherosclerosis, and contributes to vessel calcifications in T2D patients, thus suggesting an its possible role in progression of atherosclerotic lesions of other vascular beds such as lower extremity PAD of patients affected by diabetes [17, 18]. At this regard clinical studies investigating the circulating levels of OPG in patients with PAD have given controversial results [19], showing some of them no serum OPG increase in patient with PAD [20, 21], and others a positive correlation between OPG circulating levels and PAD occurrence [16, 22–24]. However currently the role of OPG and its relationship with chronic inflammatory markers in T2D patients affecting by PAD remains unclear, largely unexplored in experimental and clinical investigations, considering that there are some frequently ignored aspects like polyvascular atherosclerosis which might influence OPG levels [19].

The aims of our current study is to investigate the involvement of serum HMGB-1, OPG, HsCRP, TNF-alpha, IL-6 levels on the presence and severity of PAD in patients with T2D.

## Methods

### Study population

Diabetic patients with PAD (PAD) and diabetic controls without PAD (WPAD) were recruited among subjects consecutively admitted to the Department of Internal Medicine of the “A. Gemelli” Catholic University Hospital of Rome, Italy and to the Department of Medicine of the “St. M. Goretti” Hospital, Latina, Italy, from November 1, 2013, to January 30, 2017. Inclusion criteria for diabetic PAD patients were Caucasian race and presence of PAD at Fontaine’s stage II, III, or IV. Diagnosis of PAD was performed according to previous criteria established by the Ad Hoc Committee on Reporting Standards of the Society for Vascular Surgery and the International Society for Cardiovascular Surgery [25, 26]. All PAD patients enrolled in the study (n = 569) underwent bilateral high-resolution B-mode ultrasonography evaluation (Eco-color-Doppler Acuson 128XP/10, Acuson, Mountain View, CA, USA, with an 4 MHz transducer) and had an ABI lower than 0.8 and. In according to the Fontaine’s staging system, the severity of PAD was defined in stage II when the patients presented claudicatio intermittens, stage III when they presented rest pain, and in stage IV when ischemic trophic lesions of the lower limbs were present [27]. Following the recommendations of the Inter-Society Consensus for the Management of Peripheral Arterial Disease (TASC II), patients with ischemic rest pain, ulcers, or gangrene, attributable to objectively proven PAD, were considered affected by critical limb ischemia (CLI) [28]. Inclusion criteria for diabetic patients WPAD were Caucasian race and absence of PAD evaluated with ankle-brachial-index (ABI) and bilateral high-resolution B-mode ultrasonography. Eight hundred twenty-four diabetic subjects WPAD matched for age and gender were enrolled as controls, with an ABI ≥1 and normal findings at bilateral high-resolution B-mode ultrasonography evaluation.

Exclusion criteria from the study for whole diabetic population (with and without PAD) were liver disease, cancer, serous membrane chamber fluid, hypothyroidism, severe edema and osteoporosis. No one of the patients was taking estrogen supplements, immunosuppressive drugs, thyroxin, glucocorticoids, bisphosphonates and anticoagulants.

The presence of T2D has been confirmed by glycated hemoglobin levels >5.8%, fasting blood glucose >126 mg/dL or by indication for insulin or anti-diabetic medicaments. Hypertension has been defined as systolic blood pressure >130 mmHg, diastolic blood pressure >85 mmHg or indication to antihypertensive drugs. Hypercholesterolemia has been defined for having serum cholesterol >220 mg/dL or use of hypocholesterolemic medications. Nonsmokers were indicated patients that had never smoked or had stopped smoking within ≥1 year before enrollment in the study. All remaining patients were classified as smokers.

Approval for this study was provided by the Ethics Committees of the “A. Gemelli” Catholic University Hospital of Rome, Italy, and “St. M. Goretti” Hospital, Latina, Italy. Informed consent was obtained from enrolled patients.

### Biochemical measurements

For every patient, fast glucose, triglycerides, total cholesterol, low and high-density lipoprotein, white blood cell count have been determined. Blood samples were collected from all individuals involved after an overnight fast. Serum obtained and separated by centrifugation of blood samples was stored at −80 °C before every measurement. Serum HMGB-1 level has been determined by a commercially available ELISA kit (HMGB-1 ELISA kit II; Shino-Test Corporation, Tokyo) according to its protocol. The detection limit for HMGB-1 was 0.2 ng/mL with an inter-assay coefficient of variation (CV) <10%. HsCRP levels have been determined by using a high-sensitivity ELISA kit (Biocheck Laboratories, Toledo, OH, USA). As a capture antibody we used a monoclonal mouse anti-human OPG antibody and a biotinylated polyclonal goat antihuman OPG antibody (R&D systems). The intra and inter-assay coefficients of variation were 3.6 and 10.6%, respectively. The sensitivity, defined as the mean ± 3 SD of the 0 standard, was calculated to be 0.15 pmol/mL. By using the Quantikine ELISA kit (R&D Systems, Minneapolis, MN, USA) we have assessed serum IL-6 and TNF-alpha levels. For each patient, the serum levels were measured twice and the results were averaged.

### Statistical analysis

Demographic and clinical data between the groups have been compared using Chi squared and t tests. HMGB-1, OPG, HsCRP, TNF-alpha and IL-6 serum levels have been compared through Mann–Whitney test. Using a multivariate stepwise logistic regression analysis, two models have been tested. The first one is adjusted for traditional risk factors (all parameters are shown in Table 1), while HMGB-1 and OPG are have been included for testing in the second model. All analyses are performed using the STATA version 11.0 for Windows (Statistics/Data Analysis, Stata Corporation, College Station, TX, USA). Statistical significance has been established at *P* < 0.05.

**Table 1:** Demographic and clinical data of diabetic subjects with and without PAD

	WPAD	PAD	P value
	(n = 824)	(n = 569)	
Men/female (n)	490:334	344:225	0.243^†^
Age (years ± SD)	71.3 ± 4.4	70.7 ± 4.3	0.647*
Smoking (current) (%)	181 (22.0)	210 (36.9)	0.023^†^
Hypertension (%)	335 (40.7)	355 (62.4)	0.029^†^
CAD (%)	275 (33.4)	297 (52.2)	0.026^†^
Diabetes duration (years ± SD)	12.1 ± 4.1	12.7 ± 4.9	0.095*
Total cholesterol (mmol/L)	4.97 (1.21)	4.87 (1.15)	0.239*
HDL-C (mmol/L)	1.21 (1.04)	1.23 (1.02)	0.329*
LDL-C (mmol/L)	2.96 (1.02)	2.87 (0.93)	0.206*
Triglyceride (mmol/L)	2.05 (1.54)	1.87 (0.98)	0.913*
Fast glucose (mmol/L)	6.89 (1.92)	7.01 (2.65)^a^	0.856*
Glycated hemoglobin (%)	7.37 (1.21)	7.24 (1.14)	0.763*
Treatment			
Diet only (%)	95 (11.5)	63 (11.1)	0.658^†^
Oral agents (%)	452 (54.9)	308 (54.1)	0.341^†^
Insulin therapy (%)	277 (33.6)	198 (34.8)	0.290^†^
PAD			
1-Fontaine’s II (%)		281 (49.4)	
2-Fontaine’s III (%)		163 (28.6)	
2-Fontaine’s IV (%)		125 (22.0)	

Data are number (%) and standard deviation (SD)

*CAD* coronary artery disease, *HDL-C* high-density lipoprotein cholesterol, *LDL-C* low-density lipoprotein cholesterol

* Statistical test performed with Student’s t test

^†^Chi square test for categorical values

## Results

The socio-demographic and clinical characteristics of diabetic patients with PAD and WPAD are summarized in Table 1. Diabetic PAD patients had higher blood pressure values (*P* = 0.029) and higher CAD (*P* = 0.026) and were more often smokers (*P* = 0.023). There were no significant differences between groups regarding to median duration of diabetes (*P* = 0.095), fast glucose (*P* = 0.856), glycated hemoglobin (*P* = 0.763), total cholesterol (TC) (*P* = 0.239), HDL-C (*P* = 0.329), LDL-C (*P* = 0.206) and triglyceride (*P* = 0.913). Among 569 PAD patients, 63 diabetic patients (11.1%) were subjected to only diet, 308 (54.1%) were taking oral hypoglycemic agents and 198 (34.8%) were prescribed insulin. According to the Fontaine’s classification 281 patients were defined as stage II, 163 as stage III and 125 as stage IV.

**Fig. 1:**
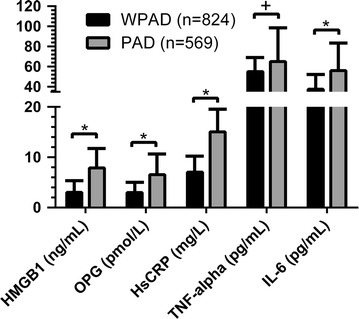
HMGB-1, OPG, HsCRP, TNF-alpha and IL-6 serum levels in diabetic patients with (PAD) and without PAD (WPAD). *HMGB-1* high-mobility group box 1, *OPG* osteoprotegerin, *HsCRP* high-sensitivity C-reactive protein, *TNF-alpha* tumor necrosis factor-alpha, *IL-6* Interleukin-6. Statistical test performed with Student’s t test. Data are shown as serum levels ± standard deviation. *P < 0.001, ^+^N.S

The diabetic PAD patients group had higher HMGB-1 (7.89 ± 15.23 ng/mL), OPG (6.54 ± 7.76 pmol/L), HsCRP (15.6 ± 14.4 mg/L) and IL-6 (56.1 ± 28.6 pg/mL) compared to WPAD group (3.02 ± 8.12 ng/mL, *P* < 0.001; 2.98 ± 2.01 pmol/L, *P* < 0.001; 7.05 ± 4.4 mg/L, *P* < 0.001; 37.5 ± 20.2 pg/mL, *P* < 0.001 respectively) (Fig. 1). No differences in serum TNF-alpha levels were detected (*P* < 0.213).

**Fig. 2:**
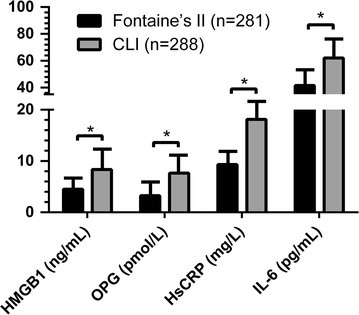
HMGB-1, OPG, HsCRP and IL-6 serum and severity of PAD (Fontaine’s II and Critical Limb Ischemia). *HMGB-1* high-mobility group box 1, *OPG* osteoprotegerin, *HsCRP* high-sensitivity C-reactive protein, *IL-6* interleukin-6. Statistical test performed with Student’s t test. Data are shown as serum levels ± standard deviation. *P < 0.001

When diabetic PAD patients were divided into two clinical categories, the first one consisting in patients affected by stable PAD (stage II, n = 281) and the second consisting in patients affected by CLI (stage III and IV, n = 288), interestingly current smoking, CAD, diabetes duration, TC and LDL-C were significantly and independently associated with CLI (Table 2). Moreover serum levels of pro-inflammatory cytokines were significantly higher in diabetic PAD patients having CLI than in those with stable PAD (HMGB-1 8.35 ± 12.72 ng/mL vs 4.53 ± 7.65 ng/mL, *P* < 0.001; OPG 7.64 ± 9.29 pmol/L vs 3.24 ± 2.56 pmol/L, *P* < 0.001; HsCRP 18.1 ± 15.9 mg/L vs 9.3 ± 5.1 mg/L, *P* < 0.001; IL-6 62.1 ± 29.4 pg/mL vs 41.5 ± 18.7 pg/mL, *P* < 0.001) (Fig. 2).

**Table 2:** Demographic and clinical data of diabetic PAD patients with Fontaine’s II and CLI

	Fontaine’s II	CLI	P value	OR (95% CI)^§^	P value
	(281)	(288)			
Men/female (n)	166:115	176:112	0.275^†^	0.91 (0.4–2.1)	0.823
Age (years ± SD)	71.9 ± 3.4	72.7 ± 4.4	0.431*	1.00 (0.9–1.00)	0.476
Smoking (current) (%)	56 (19.9)	154 (53.4)	0.001^†^	3.43 (2.15–3.65)	0.001
Hypertension (%)	173 (61.6)	182 (63.2)	0.143^†^	0.89 (0.62–1.04)	0.327
CAD (%)	102 (36.3)	195 (67.7)	0.001^†^	3.74 (2.49–4.02)	0.001
Diabetes duration (years ± SD)	9.3 ± 3.1	15.7 ± 5.1	0.001^†^	4.37 (3.07–5.12)	0.001
Total cholesterol (mmol/L)	3.65 (1.12)	5.68 (1.83)	0.001^†^	2.05 (1.54–3.02)	0.001
HDL-C (mmol/L)	1.15 (1.01)	1.31 (1.09)	0.247^†^	0.85 (0.69–1.01)	0.296
LDL-C (mmol/L)	1.95 (0.87)	3.25 (1.43)	0.001^†^	2.69 (1.32–4.12)	0.001
Triglyceride (mmol/L)	1.95 (1.45)	1.93 (1.06)	0.473^†^	1.32 (0.71–1.58)	0.456
Fast glucose (mmol/L)	6.78 (1.84)	6.97 (2.46)	0.945^†^	1.02 (0.56–1.34)	1.001
Glycated hemoglobin (%)	7.25 (1.16)	7.33 (1.32)	0.854^†^	1.00 (0.43–1.15)	0.765

Data are number (%) and standard deviation (SD)

*CAD* coronary artery disease, *HDL-C* high-density lipoprotein cholesterol, *LDL-C* low-density lipoprotein cholesterol

* Statistical test performed with Student’s t test

^†^Chi square test for categorical values

^§^Logistic regression analysis. *OR* odds ratios, *95% CI* 95% confidence intervals

The multivariate logistic regression analysis showed that in the model 1, after adjustments for cardiovascular risk factors and inflammatory cytokines, showed that smoking, hypertension, hyperlipidemia, TC, HDL-C, LDL-C, HsCRP and IL-6 were independent determinants for PAD occurrence in patients with T2D. When HMGB-1 and OPG were included in the multivariate analysis (model 2), HMGB-1 (adjusted OR 12.32; 95% CI 3.56–23.54, *P* = 0.023) and OPG (adjusted OR 3.53; 95% CI 1.54–6.15, *P* = 0.019) are resulted independently associated with PAD in T2D patients and most of the traditional cardiovascular risk factors shown in model 1 remained determinants of PAD even in model 2 (Table 3).

**Table 3:** Multivariable stepwise logistic regression model for presence of PAD

	Variable OR (95% CI)	P value
Model 1		
Age	1.02 (1.01–1.19)	0.146
Smoking	36.71 (4.54–179.31)	0.001
Hypertension	24.91 (5.56–89.65)	<0.001
Hyperlipidemia	12.54 (2.45–28.34)	0.022
TC	1.56 (1.06–1.94)	0.034
HDL-C	0.02 (0.01–0.22)	0.001
LDL-C	9.43 (1.65–74.34)	0.066
hsCRP	35.32 (7.45–129.22)	<0.001
IL-6	6.01 (2.02–16.02)	0.012
Model 2		
Sex	0.15 (0.02–0.94)	0.097
Age	1.56 (1.15–1.78)	0.164
Smoking	66.43 (4.21–654.01)	0.012
Hypertension	41.12 (6.24–189.54)	<0.001
Hyperlipidemia	8.57 (1.57–49.24)	0.017
TC	0.05 (0.01–0.43)	0.012
HDL-C	0.01 (0.00–0.24)	0.001
LDL-C	13.86 (1.34–153.27)	0.019
hsCRP	65.89 (8.11–436.53)	<0.001
IL-6	7.87 (2.47–18.04)	0.008
HMGB-1	12.32 (3.56–23.54)	0.023
OPG	3.53 (1.54–6.15)	0.019

Model 1: adjusted for traditional cardiovascular risk factors and established inflammatory cytokines; Model 2: adjusted for the risk factors in model 1 plus HMGB-1 and OPG

## Discussion

### Cross-talk between HMGB-1 and inflammatory molecules

The present study has demonstrated in a large population of T2D patients that serum HMGB-1 levels are significantly increased in PAD patients and correlated with clinical severity. Particularly we found that the HMGB-1 serum levels are statistically significant higher in T2D patients with PAD than in diabetic control WPAD (*P* < 0.001). Our observations are consistent with the study of Oozawa and collaborators, conducted in smaller population where few diabetic patients were compared to healthy controls, instead of T2D patients [29]. Our choice to investigate only diabetic patients has been suggested by the evidence that diabetes is one of the strongest predictor for critical limb ischemia occurrence [30].

Furthermore HMGB-1 serum levels increased according to the Fontaine’s stage. In fact when diabetic PAD patients were divided into two clinical categories, consisting in patients affected by stable PAD and in patients affected by CLI, HMGB-1 serum levels were significantly higher in patients affected by CLI than stable PAD (*P* < 0.001).

In addition the present study confirmed our previous data showing that OPG, HsCRP and IL-6 serum levels are associated with PAD (*P* < 0.001, *P* < 0.001 and *P* < 0.001 respectively; Fig. 1) and gradually increase according to clinical severity of disease (Fig. 2). The relationship persists significant also after adjustment for potential confounding variables such as age, smoking status, hypertension, serum lipid profile, glycaemic control, and chronic inflammation. A multivariate logistic regression analysis demonstrate that both HMGB-1 and OPG remain independently associated with CLI in diabetic patients (model 2) and most of traditional risk factors in model 1 are determinants of CLI also in model 2 (Table 3).

Our data are in agreement with previous reporting suggesting a cross-talk among HMGB-1 and inflammatory molecules, including CRP. Specifically Kawahara and coworkers demonstrated that, through the p38MAPK pathway, CRP induces the production of HMGB-1 in a dose-dependently way [8]. Moreover HMGB-1 up-regulates pro-inflammatory cytokines expression [31, 32], suggesting that this way contributes to the inflammatory process and to atherosclerotic lesion development [33, 34]. The role of chronic inflammation in the development of PAD and of its complications has been extensively investigated in the past. Malmstedt reported the role of the receptor for advanced glycation end products (RAGE), showing that higher circulating levels of an endogenous ligand for RAGE, the pro-inflammatory protein S100A12, was associated with increased risk for amputation or death and with earlier development of PAD [35]. Several circulating inflammatory biomarkers have been suggested to predict occurrence (i.e. Rho-kinase activity, human cartilage glycoprotein-39, TWEAK, PON-3, B2M, NO, NOX-2, TGF-β1, TSP-1, VEGF) and/or the severity of PAD (i.e. sVCAM-1, MPO, NT-proBNP, VEGF, VEGF-A isoforms and MMPs). Anyway their validity as reliable clinical markers is still debated [36].

Two sources have been proposed to increase HMGB-1 circulating levels, the local HMGB-1 release promoted by damaged immune cells such as monocytes, macrophages, endothelial cells cytokine-triggered, or the secretion from vascular smooth muscle cells (VSMCs) in atherosclerotic lesions [5, 37]. Moreover VSMCs may be both source and target of HMGB-1 having been shown VSMCs proliferation following HMGB-1 self-secreted by them. This mechanism could explain our observation that HMGB-1 serum levels strongly correlate with PAD severity. In fact, as atherosclerosis goes over, more macrophages releasing HMGB-1 are recruited and induce proliferation and migration of VSMCs that in turn increase their self-secretion of HMGB-1 stimulating further their proliferation. This mechanism self-perpetuating can contribute to progression of atherosclerotic lesions [33]. Moreover HMGB-1 mRNA expression could be strongly up-regulated by inflammatory cytokines such as TNF-alpha [33]. In our study we have confirmed that serum levels of TNF-α, as well IL-6 and HsCRP, are significantly increased in diabetic patients with concomitant PAD [38, 39]. Similarly in animal studies HMGB-1 has been shown play an important role in atherosclerotic process exerting pro-atherogenic effects through the modulation of pro-inflammatory mediators, macrophage migration, and accumulating smooth muscle and immune cells. HMGB-1 neutralization also reduces diet-induced atherosclerosis in apolipoprotein E-deficient mice [40]. Moreover high HMGB-1 serum levels have been reported be associated with CAD in patients with and without T2D and they were gradually correlated with the severity of coronary artery stenosis [41, 42].

### OPG and its role in endothelial dysfunction

HMGB-1 is a bone-active cytokine and on multiple murine bone cells culture recombinant protein (rHMGB-1) enhanced the RANKL/OPG steady state mRNA ratio and augmented the release of TNF-alpha and IL-6 in osteoblastogenic bone marrow stromal cell (BMSC) [15]. In our study we also measured circulating levels of OPG establishing a positive correlation between serum OPG levels and the occurrence of PAD in diabetic patients (Fig. 1). Moreover OPG has resulted a significant predictor of disease severity (Fig. 2).

The association between OPG and atherosclerosis in CAD is quite well studied. OPG has been reported an independent risk factor for development and progression of atherosclerosis in patients with CAD [43]. Also OPG predicted early carotid atherosclerosis in patients with CAD [44]. In our study, as shown in Table 1, CAD occurrence is similar between the two groups (*P* = 0.294), thus it is to be ruled out it as a possible confounder. Studies investigating the role of OPG in PAD have done contrasting and uncertain results. While some of them did not find any serum OPG level increase in patient with PAD [19, 20], others showed a positive correlation between circulating OPG and severity of PAD [18] or reported OPG as an independent predictor of PAD [33]. More recently two studies investigated the association between OPG and PAD in T2D patients [45, 46] and demonstrated that OPG concentrations are significantly increased in T2D patients having PAD in comparison with T2D without PAD. However they have focused on a smaller and younger population of patients, and no data were available regarding CAD occurrence as in our report. A recent our report showed a significant positive association between OPG serum levels and occurrence and severity of PAD in a population of T2D [23]. However in this study, patients affected by PAD also had a significantly higher occurrence of CAD compared to diabetic patients without PAD, so it could not be ruled out as possible bias to investigations. Of interest, we also reported for the first time that fibroblast growth factor 23 (FGF23) is a predictor of PAD occurrence and severity, moving from a previous report of our group [47] in which we suggested the association between FGF23 and OPG in patients with unstable carotid plaques, thus underlying the role of the two molecules in the development of atherosclerosis owing to their contribute to vascular calcification. To corroborate the involvement of FGFs family members in the early stage of atherosclerosis, Zhang’s group [48] observed that circulating FGF21, an emerging metabolite regulating glucose and lipid metabolism, is directly associated with lower extremity atherosclerosis disease. They also found an association between serum FGF21 levels and carotid intima thickness in the female group, accounting maybe for a gender-specific effect. All together these studies took in account the endothelial dysfunction, an early and important trigger of atherogenesis, as mechanism responsible for PAD development and worsening. In this direction an interesting significant association of high OPG was found with the non-0 blood groups in PAD patients, considering that non-0 blood group subjects have a well-established higher risk of developing thrombotic episode of venous or arterial origin [49].

Whether OPG plays a causal role in mediating or protecting against vascular injury is presently unclear. An atheroprotective effect of OPG could be related to its anti-calcification function or due to its action as a decoy receptor for the receptor activator of nuclear factor-B ligand (RANK-L) and the TNF-related apoptosis inducing ligand (TRAIL) by which it blocks their subsequent pro-inflammatory and pro-apoptotic effects [50, 51]. In particular OPG has been identified as an in vitro survival factor for endothelial cells [52]. On the other hand recent evidence supports a pro-atherosclerotic role for OPG including its capacity to enhance the expression of endothelial cell adhesion molecules promoting infiltration of leukocytes and monocytic cells [53]. However, there are some frequently ignored aspects like polyvascular atherosclerosis which might influence OPG levels [19]. Furthermore, OPG might contribute to endothelial dysfunction by reducing the nitric oxide synthase protective pathway by blocking RANK-L [54]. A longitudinal prospective study is needed to clarify the causal relationship among HMGB-1, OPG and PAD.

### Limitations

There are some limitations in the present study. First, we developed a case–control study, thus survival and enrollment bias should be considered. Second, we collected data from an European cohort having other cardiovascular diseases. Also, comorbidities might represent confounding factors and our findings are not generalizable to other age groups or ethnicities. So our observations need to be confirmed in larger populations and different ethnic groups. Finally the influence of other pro-inflammatory molecules such as myeloperoxidase, soluble adhesion molecules, sCD40 and matrix metalloproteinases did not evaluate in this study.

## Conclusions

The present study demonstrated that HMGB-1 and OPG serum levels are statistically and independently associated with occurrence and clinical severity of PAD in an Italian population affected by T2D. The understanding of pathophysiological mechanisms and of molecular pathways responsible for HMGB-1 and OPG involvement in atherosclerosis disease may provide us new therapeutic weapons to manage diabetic complications and morbidities. Furthermore the measurement of these two serum markers could be useful as diagnostic and prognostic markers of PAD in diabetic patients.

## Abbreviations

ABI: ankle-brachial-index
BMSC: bone marrow stromal cell
B2M: beta 2 microglobulin
CAD: coronary artery disease
CLI: critical limb ischemia
FGF21: fibroblast growth factor 21
FGF23: fibroblast growth factor 23
HDL-C: high-density lipoprotein cholesterol
HMGB-1: high mobility group box-1
HsCRP: high sensitive C-reactive protein
ICAM-1: intercellular adhesion molecule-1
IL-6: interleukin-6
LDL-C: low-density lipoprotein cholesterol
MMPs: matrix metalloproteinases
MPO: myeloperoxidase
NO: nitric oxide
NOX-2: nicotinamide adenine dinucleotide phosphate oxidase-2
NT-proBNP: N-terminal pro-brain natriuretic peptide
OPG: osteoprotegerin
OR: odds ratio
PAD: peripheral arterial disease
PON-3: paraoxonase-3
RAGE: receptor for advanced glycation end products
rHMGB-1: recombinant protein HMGB-1
SD: standard deviation
sVCAM-1: soluble intercellular adhesion molecule 1
TC: total cholesterol
TGF-β1: transforming growth factor-β1
TNF-alpha: tumor necrosis factor-alpha
TSP-1: thrombospondin-1
T2D: type 2 diabetes
TWEAK: tumor necrosis factor-related weak inducer of apoptosis
VEGF: vascular endothelial growth factor
WPAD: without peripheral artery disease
